# T‐Cell Populations in Infancy After Maternal Probiotic Supplementation to Prevent Atopic Dermatitis

**DOI:** 10.1002/clt2.70161

**Published:** 2026-02-26

**Authors:** Dinastry Pramadita Zakiudin, Anne Dorthea Bjerkenes Rø, Vibeke Videm, Gunnhild Vatne Leirvik, Marte Høen Lein, Torbjørn Øien, Melanie Rae Simpson

**Affiliations:** ^1^ Department of Public Health and Nursing NTNU ‐ Norwegian University of Science and Technology Trondheim Norway; ^2^ Clinic for Laboratory Medicine St. Olavs Hospital Trondheim University Hospital Trondheim Norway; ^3^ Department of Clinical and Molecular Medicine NTNU ‐ Norwegian University of Science and Technology Trondheim Norway; ^4^ Department of Immunology and Transfusion Medicine St. Olavs Hospital Trondheim University Hospital Trondheim Norway

**Keywords:** atopic dermatitis, children, probiotic, T cells, Th22

## Abstract

**Background:**

In the randomised, controlled study Probiotics in the Prevention of Allergy amongst Children in Trondheim (ProPACT), maternal probiotics given from 36 weeks pregnancy until 3 months post‐delivery while breastfeeding reduced atopic dermatitis (AD) in the offspring. Previous analysis of T helper (Th) subsets indicated that the preventive effect may be partially mediated through reduced Th22 percentage at 3 months of age.

**Objective:**

To examine the longitudinal effects of maternal probiotics on Th1, Th2, Th17, Th22, and regulatory T cells (Treg) in offspring at 10 days and 2 years of age compared to the previously published 3 months results.

**Methods:**

Pregnant women (*n* = 415) were randomised to take probiotic milk (*Lacticaseibacillus rhamnosus GG*, *Bifidobacterium animalis subsp*. *lactis Bb‐12* and *Lactobacillus acidophilus La‐5*) or placebo, and their offspring were assessed for AD at 2 years. We analysed the children's blood collected at 10 days (*n* = 112) and 2 years (*n* = 156) for Treg and Th subsets using flow cytometry and included the results from the previously analysed 3 months samples (*n* = 76) in the same study to compare the three timepoints using linear mixed models.

**Results:**

There were no statistically significant differences between T cell populations of the children in the probiotics and placebo groups at 10 days and 2 years.

**Conclusion:**

We previously observed reduced Th22 percentage in the probiotics group at 3 months. However, since the effect was not seen earlier and did not last, it may not be the main reason for AD prevention.

## 1 Introduction

Deviations in immune system development in early infancy may lead to immune‐related disorders [1] such as atopic dermatitis (AD) which often presents before 5 years of age [2, 3]. Maternal probiotics may potentially promote long‐term health of the offspring [4] and the use of probiotics in the prevention of AD has shown particular promise [5]. However, the mechanisms behind the preventive effect are incompletely understood.

T helper (Th) cells and regulatory T cells (Treg) are known to play an important role in the presence and severity of AD. Probiotics given to pregnant mothers and their infants have been associated with a skew towards a Th1 profile with an increase in the Th1‐promoting cytokine interferon gamma (IFNγ) [6]. Other studies have reported no effect on either the Th1/Th2 balance or Treg of the infants [6, 7]. Similarly, we have previously found no differences observed in Th1, Th2, Th17, or Treg in 3‐month‐old infants after maternal probiotic supplementation [8]. There was, however, a reduction in the proportion of Th22 cells, which was also found to potentially play a role in preventing AD in offspring from probiotic supplemented mothers [8]. In the current study, we analysed T cell population patterns from peripheral blood mononuclear cells (PBMC) at 10 days and 2 years of age using multicolour flow cytometry. Together with the previously published analyses of samples collected at 3 months in the same study we evaluate the longitudinal effect of maternal probiotics on T cell subsets (Th1, Th2, Th17, Th22, Treg) in their offspring.

We hypothesised that AD prevention after maternal probiotic supplementation is partially due to changes in T cell populations. We postulated that the reduction in Th22 previously seen at 3 months [8] would be apparent as early as 10 days of age and persist until 2 years of age. Our primary aim in this study was to examine whether probiotics influenced T cell population development in children earlier and later than 3 months until 2 years of age. As our secondary aims, we investigated the association between T cell populations and the presence and severity of AD and examined the general development of T cell populations in children from 10 days to 2 years of age.

## 2 Methods

### 2.1 Participants and Sample Collection

In the ProPACT study, 415 pregnant women were randomised to receive probiotics or placebo milk from 36 weeks of gestation until 3 months post‐delivery while breastfeeding [9, 10]. The pregnant women were recruited from a non‐selected population, and a computer‐generated randomisation sequence allocated them to probiotic or placebo milk. The probiotic milk corresponded to a daily dose of 5 × 10^10^ colony‐forming units (CFU) *Lacticaseibacillus rhamnosus GG* (LGG), 5 × 10^10^ CFU *Bifidobacterium animalis subsp*. *Lactis Bb‐12* (Bb‐12) and 5 × 10^9^ CFU *Lactobacillus acidophilus La‐5* (La‐5), whilst the placebo was fermented and pasteurised skim milk with similar taste without probiotic bacteria.

Information regarding demographics and risk factors for allergy‐related diseases was obtained from questionnaires completed during pregnancy, and at the ages of 6 weeks, 1 year and 2 years. Mothers were encouraged to bring their children to an examination by a trained nurse if they had an itchy rash for more than 4 weeks at any time during the first year of life to ensure that all AD cases were identified. A paediatrician examined all children at 2 years of age, and AD was defined using the U.K. working party's diagnostic criteria for AD [11]. AD severity was assessed with the Nottingham Eczema Severity Score (NESS) [12]. Allergic sensitisation was defined as either a positive skin prick test (SPT) or elevated specific IgE (≥ 0.35 kU L^−1^) [13]. Asthma was defined as at least three episodes of wheezing in the last 12 months combined with treatment by inhaled glucocorticoids, or signs of suspected hyper‐reactivity (cough or wheeze at excitement or impaired night sleep) without concurrent upper respiratory infection. All participating mothers signed a written consent form. The study was approved by the Regional Committee for Medical Research Ethics in Central Norway (097‐03; 2012/2123) and registered at ClinicalTrials.gov (NCT00159523).

**FIGURE 1:**
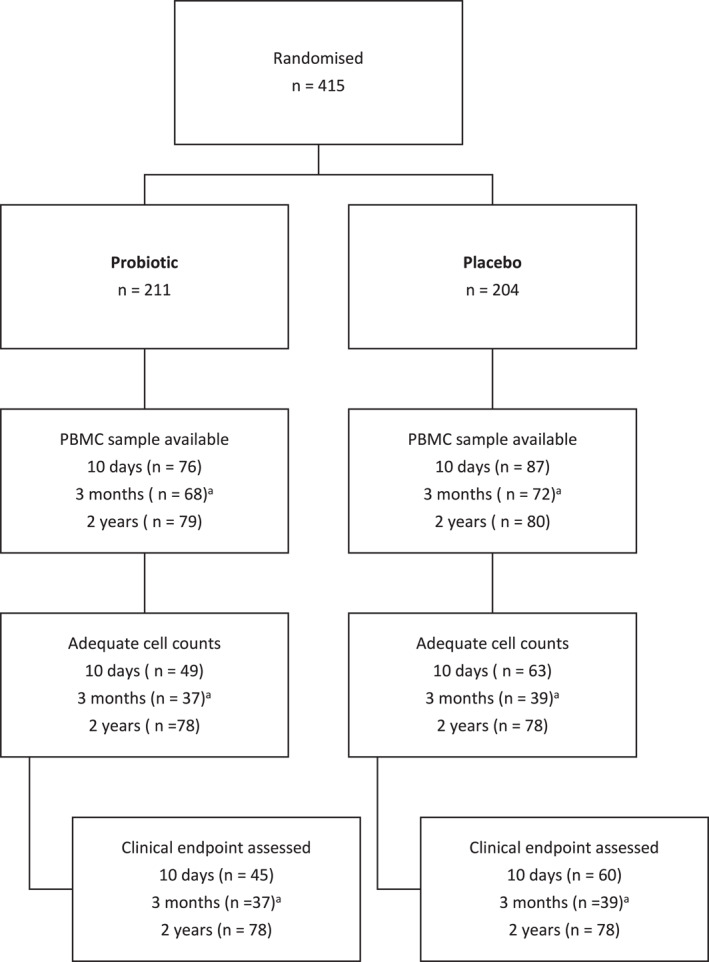
Participant inclusion and available blood samples of children. ^a^Samples collected at 3 months have been previously analysed and published, and the results from Th cell subsets are presented together in this longitudinal study. The number samples with adequate cell counts at 3 months represents the number with adequate cells to complete Th cell analyses following a protocol that prioritised Treg cell analyses first.

PBMCs were a useful and feasible sample type for this longitudinal study in children, as they allowed for the isolation of T cells from small blood volumes across multiple time points, minimising invasiveness despite the repeated sampling required. Children who attended the clinical follow‐up at 10 days and 2 years and had at least one available PBMC sample with adequate cell count for analysis, were eligible for inclusion in this study. The previously published results from the PBMC samples collected at 3 months were included in the novel statistical analysis, as detailed below. Ultimately, 220 children had at least one measurement of T cell subsets and could be included in the study. A total of 112 children had data at 10 days, 76 children from the previous study had data at 3 months [8], and 156 children had data at two years (Figure 1).

### 2.2 Cell Analyses

Samples from each timepoint were analysed at separate times using slightly different protocols for the intracellular staining of the T cells. Within each timepoint, the samples were analysed in smaller batches over a short period of time. The initial thawing, incubation and cell stimulation was identical for samples collected at all three timepoints. Frozen PBMCs samples from 10 days to 2 years were thawed and incubated overnight at 10^6^cells/mL in RPMI‐1640 medium (Sigma Aldrich, St. Louis, MO, USA) with 10% inactivated Foetal Bovine Serum (FBS) (Sigma Aldrich, St. Louis, MO, USA). The cells were plated into 24‐well flat‐bottom plates and incubated in a 37°C humified atmosphere containing 5% CO_2_. The next day, Cell stimulation Cocktail (phorbol 12‐myristate 13‐acetate (PMA), ionomycin, brefeldin A and monensin) Plus Transport Inhibitors (500x) (eBioscience, Carlsbad, CA, USA) was diluted with 10% FBS RPMI and added to each well. The cells were further incubated for 5 h at 37°C. Immediately after stimulation, the cells were analysed using multicolour flow cytometry involving a three‐step protocol: viability, surface, and intracellular staining (further details in the Supporting Information S1). For each sample, a minimum of 10,000 live cell events were collected.

An 8‐colour antibody panel was used for 10‐day and 2‐year samples and included: CD3 fluorescein isothiocyanate (FITC)/IL‐4 Phycoerythrin (PE)/IL‐10 Peridinin Chlorophyll Protein Complex (PerCP)‐eFluor710/IFNƴ PE‐Cyanine (Cy‐7)/IL‐22 eFluor660/IL‐17 Allophycocyanin (APC)‐eFluor780/CD4 eFluor450/Fixable viability dye (FVD) eFluor506. The analysis methods used for the 3‐month samples have been described previously [8]. Briefly, the primary difference is in the analysis of Treg which were identified using a separate panel and intracellular staining with CD127 PerCP‐Cy5.5/FoxP3 PE/FVD 506 rather than extracellular staining for IL‐10 as used for the 10‐day and 2‐year samples.

For data analysis, we used the Infinicyt software (v 2.0 Cytognos S.L., BD, Salamanca, Spain). Following gating of live cells as those excluding FVD 506 on a side scatter versus FVD plot, Th cells were gated as CD3+ and CD4+, and doublets were removed. The Th cell subsets were then defined as follows: Th1 cells: IFNγ+ IL‐4‐ and Th2 cells: IFNγ‐ IL‐4+ in a IFNγ versus IL‐4 plot, Th22 cells: IL‐17‐ IL‐22+ in an IL‐17 versus IL‐22 plot, Th17 cells: IL‐17+ IFNγ‐in an IL‐17 versus IFNγ plot, and Treg cells as IL‐10+ IFNγ‐in an IL‐10 versus IFNγ plot. As previously published [8], Treg cells had been gated as intracellular FoxP3+/CD25^hi+^/CD127^lo/‐^ in the 3 months samples. This procedure was not continued because it necessitated two tubes for complete characterisation, which led to incomplete data due to low cell numbers in several samples.

Fluorescence‐minus‐one (samples stained with all antibodies except the one of interest) and internal cell populations were used as controls. Positive events were defined as positive above the level of controls. Proportions of Th cell subsets are given as percentages of the total Th cell population.

### 2.3 Statistics

We used STATA 18 (StataCorp, College Station, TX, USA) for statistical analysis which included the new data from 10 days to 2 years, together with a re‐analysis of the data from 3‐month samples. Data are given as percentages for categorical data and mean and standard deviation or median with interquartile range (IQR) for continuous data as appropriate. Differences in Th cell proportions between the probiotics and placebo groups were analysed using linear mixed regression models (LMM) with participant ID as a random effect, and the fixed effects included probiotics, age, and an interaction term between probiotics and age. A separate regression analysis was completed for each T cell subset. During data analysis it became apparent that the differences in Treg staining were not comparable enough to include in the same regression analyses, and the model for Treg therefore included only 10‐day and 2‐year samples. The distribution of the observed T cell proportions and the residuals from the regression analyses based on these observed values were highly right skewed. The presented analyses are therefore based on T cell proportions which have been log‐transformed using the natural logarithm. The non‐parametric Mann‐Whitney *U*‐test was employed as a sensitivity analysis to compare groups for each T cell subtype and at each timepoint separately. The same linear mixed regression strategy was used to assess the association between T cell populations and the presence of AD and AD severity category. When analysing the association between T cell subtypes and severity, cases were classified using the NESS score as mild (score: 3–8), moderate (score: 9‐11) or severe (score: 12‐15). Two‐sided *p*‐values < 0.05 were considered statistically significant. The development of T cell proportions over time are presented descriptively, without formal statistical comparison due to the potential for assay related variation between timepoints.

## 3 Results

PBMCs samples collected from children at 10 days and 2 years were analysed in this study together with the results from samples collected at 3 months which have been published in an earlier study (Figure 1). The characteristics of the study population who had at least one available measurement of Th cell subsets are summarised in Table 1. The probiotics group had a higher proportion of male children and children with older siblings compared to the placebo group. Consistent with the overall results from the ProPACT study, the probiotics group in these analyses had a lower cumulative incidence of AD, as well as a lower current prevalence at 2 years of AD and a lower proportion with current asthma (Table 1).

**TABLE 1:** Baseline characteristics of study population with at least one measurement of T cell subsets (*n* total samples = 220).

Baseline characteristic	Probiotic (*n* = 107)		Placebo (*n* = 113)		Total (*n* = 220)	
	*N*		*N*		*N*	
Sex, male, *n* (%)	106	55 (51.9)	112	42 (37.5)	218	97 (44.5)
Birthweight, g, mean (SD)	106	3688 (482)	111	3584 (506)	217	3632 (496)
Maternal age, years, mean (SD)	103	30.5 (3.6)	110	30.6 (3.9)	213	30.5 (3.8)
Born > 2 weeks before term, *n* (%)	99	8 (8.1)	104	7 (6.7)	203	15 (7.4)
Maternal atopy, *n* (%)	107	48 (44.9)	111	63 (56.8)	218	111 (50.9)
Atopy in the family, *n* (%)	107	70 (65.4)	112	78 (69.6)	219	148 (67.6)
Antibiotics first year, *n* (%)	103	16 (15.5)	105	18 (17.1)	208	34 (16.4)
Maternal smoking, *n* (%)	104	3 (2.9)	110	3 (2.7)	214	6 (2.8)
Older sibling, *n* (%)	107	49 (45.8)	111	47 (42.3)	218	96 (44.0)
Pets in the house, *n* (%)	107	26 (24.3)	112	33 (29.5)	219	59 (26.9)

Allergy‐related disease at 2 years	*N*	*n* (%)	*N*	***n* (%)**	**RR (95% CI)**	***p*‐val**
Cumulative AD, *n* (%)	103	21 (20.4)	110	38 (34.5)	0.59 (0.37–0.94)	0.021
Severity of AD						
Mild AD	21	18 (85.7)	38	23 (60.5)		0.08
Moderate AD		2 (9.5)		14 (36.8)		
Severe AD		1 (4.8)		1 (2.6)		
Sensitivity (IgE/skin prick pos.), *n* (%)	98	15 (15.3)	105	12 (11.4)	1.34 (0.66–2.72)	0.416
Current AD, *n* (%)	103	10 (9.7)	110	14 (12.7)	0.76 (0.35–1.64)	0.486
Current asthma	103	4 (3.9)	110	10 (9.1)	0.43 (0.14–1.32)	0.125

Abbreviations: AD: Atopic Dermatitis; IgE: Immunoglobulin E; SD: Standard Deviation.

### 3.1 T Cell Populations and Probiotic Supplementation

The observed proportions of T cell subtypes for the probiotics and placebo groups and the estimated difference between the groups at each timepoint are presented in Table 2.

**TABLE 2:** Observed proportion of T cell subsets in probiotic and placebo groups and estimated difference between groups.

Time	Probiotic			Placebo			Probiotic versus Placebo^a^	
	*N*	Mean %(SD)	Median % (IQR)	*N*	Mean % (SD)	Median % (IQR)	Estimated fold‐change (95% CI)	*p*‐value
Th1								
10 days	49	0.52 (0.32)	0.42 (0.33–0.61)	63	0.53 (0.40)	0.45 (0.32–0.64)	1.00 (0.78–1.28)	1.00
3 months^b^	37	0.99 (0.55)	0.80 (0.60–1.19)	39	0.93 (0.52)	0.81 (0.53–1.14)	1.07 (0.79–1.44)	0.67
2 years	78	3.38 (4.11)	2.47 (1.04–4.43)	78	3.57 (3.19)	3.11 (1.57–4.15)	0.86 (0.69–1.07)	0.17
Th2								
10 days	49	0.40 (0.27)	0.35 (0.22–0.50)	63	0.36 (0.22)	0.30 (0.19–0.49)	1.11 (0.81–1.52)	0.52
3 months^b^	37	0.24 (0.12)	0.23 (0.14–0.33)	39	0.21 (0.10)	0.19 (0.14–0.27)	1.11 (0.76–1.63)	0.59
2 years	78	5.81 (7.67)	2.99 (1.46–6.33)	78	7.80 (11.61)	3.05 (1.74–7.50)	0.93 (0.71–1.21)	0.58
Th17								
10 days	49	0.11 (0.09)	0.10 (0.04–0.16)	63	0.11 (0.09)	0.09 (0.05–0.14)	0.92 (0.64–1.31)	0.64
3 months^b^	37	0.22 (0.17)	0.21 (0.08–0.30)	39	0.17 (0.12)	0.14 (0.06–0.21)	1.22 (0.80–1.88)	0.36
2 years	78	0.16 (0.52)	0.07 (0.01–0.15)	78	0.11 (0.11)	0.07 (0.02–0.16)	0.96 (0.68–1.35)	0.81
Th22								
10 days	49	0.02 (0.01)	0.01 (0.01–0.02)	63	0.02 (0.02)	0.01 (0.01–0.03)	0.86 (0.62–1.19)	0.37
3 months^b^	37	0.06 (0.06)	0.04 (0.03–0.06)	39	0.07 (0.04)	0.06 (0.04–0.09)	0.73 (0.49–1.08)	0.11
2 years	78	0.05 (0.12)	0.02 (0.00–0.06)	78	0.06 (0.23)	0.02 (0.00–0.05)	1.17 (0.84–1.64)	0.35
Treg								
10 days	49	0.05 (0.03)	0.04 (0.03–0.06)	63	0.06 (0.04)	0.04 (0.03–0.06)	0.97 (0.77–1.23)	0.88
2 years	78	0.18 (1.00)	0.02 (0.00–0.03)	78	0.06 (0.26)	0.02 (0.00–0.05)	1.00 (0.85–1.17)	1.00

Abbreviations: CI: Confidence Interval; IQR: inter‐quartile range; SD: Standard Deviation.

^a^ Differences between the probiotic and placebo groups at each timepoint were estimated using linear mixed regression models on log‐transformed T subset proportions and the estimated coefficients are therefore fold‐change estimates.

^b^ The results from the previously published results from samples collected at 3 months were also included in this longitudinal study.

**FIGURE 2:**
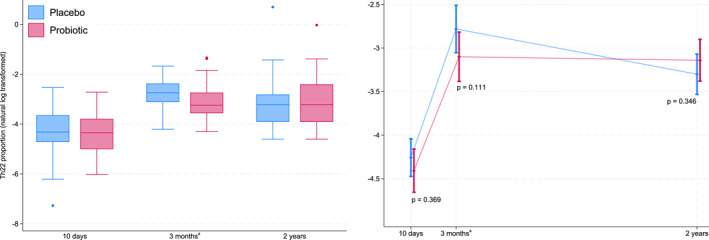
Log‐transformed proportions of Th22 cells (left) and estimated means and 95% CI from linear mixed model (right). Blue: placebo group. Red: probiotic group. ^a^The results from the previously published analyses of samples collected at 3 months were also included in this longitudinal study.

Negligible differences were observed between the probiotics and placebo groups, except for Th22 cells which, as described previously, were marginally lower in the probiotics group compared to the placebo group (median 0.04% vs. 0.06%). Whilst this reduction was not statistically significant in the linear mixed regression results, the non‐parametric Mann‐Whitney *U*‐test replicated our previous findings (Supporting Information Table S1). There was no conclusive indication that the reduction in Th22 was present earlier at 10 days, or was persistent at 2 years (Table 2, Table S1 and Figure 2).

### 3.2 T Cell Populations and Atopic Dermatitis

**FIGURE 3:**
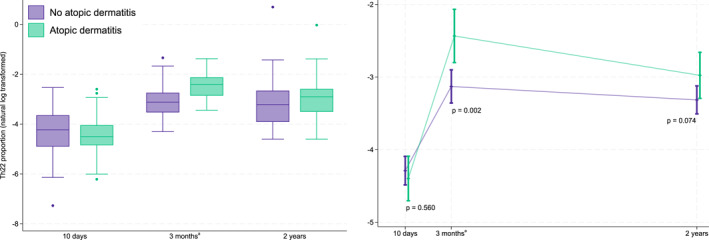
Log‐transformed proportions of Th22 cells (left) and estimated means and 95% CI from linear mixed model (right). Purple: children without AD. Green: children with AD. ^a^ The results from the previously published analyses of samples collected at 3 months were also included in this longitudinal study.

The observed proportions of T cell subtypes for the children with AD and without AD and the estimated difference between the groups at each timepoint are presented in Supporting Information Table S2. Overall, there was a tendency at 2 years that children with AD had higher Th1, although this was not statistically significant (estimated fold‐change for Th1: 1.26, 95% CI 0.98 to 1.61, *p* = 0.07). There were no clear differences in Th17 cells and Treg between children with and without AD (Table S2). We observed close to significantly higher Th22 cells in 3‐month‐ and 2‐year‐old children (estimated fold‐change at 2‐year: 1.40, 95% CI 0.97 to 2.03, *p* = 0.07). The Th22 cell proportion tends to increase from 10 days to 3 months both in children with and without AD, but with a greater increase amongst those with AD. By 2 years, both those with and without AD had similar levels of Th22 cells again (Figure 3).

### 3.3 T Cell Populations and Atopic Dermatitis Severity

**FIGURE 4:**
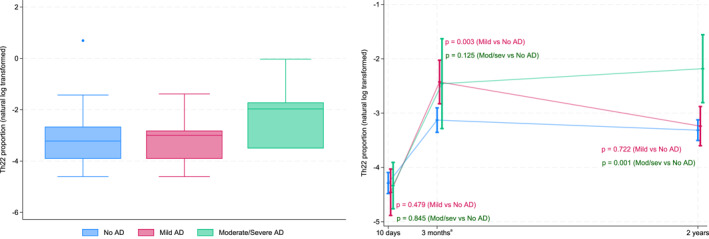
Log‐transformed proportions of Th22 cells (left) at 2 years of age based on AD severity and estimated means and 95% CI from linear mixed model (right). Blue: children without AD. Pink: children mild AD. Green: children with moderate or severe AD. ^a^ The results from the previously published analyses of samples collected at 3 months were also included in this longitudinal study.

The observed proportions of T cell subtypes in relation to AD severity and the estimated difference between the groups at each timepoint are presented in the Supporting Information Table S3. In children with mild AD, we observed a higher proportion of Th22 cells at 3 months of age compared to those without AD (estimated fold‐change: 2.01, 95% CI 1.30 to 3.09, *p* = 0.002, Figure 4, Table S3). There were no clear differences in other Th subsets or Treg although we cannot rule out an association considering the wide confidence intervals for all comparisons (Table S3).

In children with moderate or severe AD compared to children without AD, we observed higher Th22 cells at the age of 2 years (estimated fold‐change: 3.10, 95% CI 1.61 to 5.96, *p* < 0.001, Figure 4, Table S3). Similarly, Th22 was higher in these children at 3 months of age, although the difference was not statistically significant (estimated fold‐change: 1.96, 95% CI 0.83 to 4.61, *p* = 0.12). At 2 years, Th1 cells were higher (estimated fold‐change: 1.79, 95% CI 1.14 to 2.83, *p* = 0.01, Table S3). Figure 4 shows Th22 cells proportions at different timepoints and Th22 cell proportions according to the AD severity level of the children.

### 3.4 T Cell Development Over Time

**FIGURE 5:**
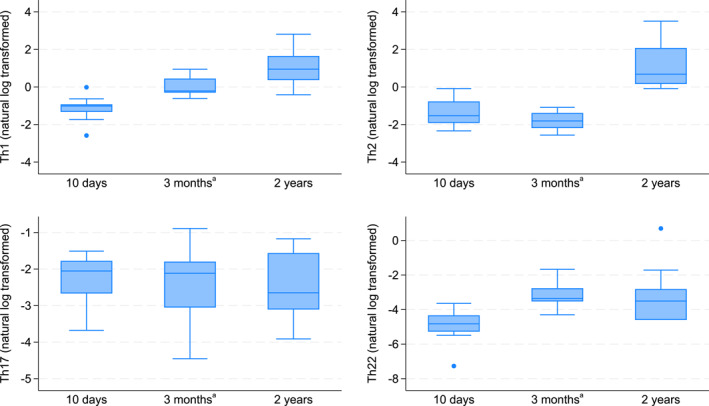
Log‐transformed T helper cell proportions over time for all children. ^a^The results from the previously published analyses of samples collected at 3 months were also included in this longitudinal study.

A low proportion of T cells had differentiated into Th subsets or Treg at 10 days and 3 months. The Th1 and Th2 subsets were most common, yet on average accounted for less than 1% and 0.5% of T cells at both early timepoints, respectively (Supporting Information Tables S2 and S4). Figure 5 provides a descriptive presentation of the development of T cell proportions at each timepoints.

The proportions of Th1 and Th2 cells were substantially higher at 2 years, with Th1 cell proportions rising progressively from 10 days to 3 months to 2 years and Th2 cell proportions first decreasing at 3 months before increasing again to 2 years (Figure 5, Supporting Information Tables S2 and S4). There was a relatively constant proportion of Th17 cells across the timepoints and a slight increase in Th22 cells from 10 days to 3 months followed by a decreasing trend to 2 years.

## 4 Discussion

In this study, maternal probiotic supplementation did not appear to affect the proportion of Th1, Th2, Th17, Th22 cells, and Treg cells in offspring at 10 days or 2 years, and as was shown previously, it was associated only with a slight decrease in the Th22 cell proportion at 3 months of age [8]. Otherwise, children with AD by 2 years of age had slightly higher levels of Th22 cells at 3 months of age, those with mild AD had increased proportions of Th22 cells at 3 months of age, and those with moderate to severe AD had increased proportions of Th22 cells at 2 years compared to those without AD.

Very few studies have examined T cell populations in infancy, and none have directly looked at the impact of maternal probiotic supplementation in humans. An animal study found that prenatal probiotic supplementation increased mature T cells in the pups [14]. Another study showed that prenatal *Lactobacillus reuteri* and omega‐3 supplementation led to hypermethylation, affecting immune pathways in neonatal Th cells [15]. Our findings align with previous research on T cell development in children, noting increases in Th1 and Th2 cells [16, 17], although the early decline in Th2 cells from 10 days to 3 months observed in our study has not been previously reported. The stability of Th17 cell amount in early childhood has also been described [2].

The association between maternal probiotic supplementation and T cell subsets may be limited to a slight reduction in Th22 cells at 3 months post‐birth. The reduction in Th22 was not seen at 10 days or 2 years, and no other T cell subsets differed between groups. As such, changes in Treg or Th subsets up to 2 years are unlikely to explain AD prevention, with the possible exception of a transient difference in the proportion of Th22 cells. The role of Th22 cells and IL‐22 in the immunopathology of AD has been investigated both in skin lesions and in blood samples. Atopic diseases is typically associated with Th2, however, many patients with AD have an early and ongoing upregulation of Th2/Th22 [18, 19] and Th2 primarily mediates paediatric AD [20]. Secreted by Th22 cells, IL‐22 increases keratinocyte proliferation but simultaneously inhibits their differentiation [21]. Normal keratinocyte differentiation is crucial for forming a robust stratum corneum, the outermost layer of the skin barrier. In AD, this imbalance leads to epidermal hyperplasia (thickening) without proper maturation, resulting in a structurally compromised barrier [22]. Whilst we have not measured circulating IL‐22 in these children, we have previously found lower levels of the related IL‐17C at 2 years of age in the probiotic group [23], which may also be related to reduced Th22 cells [24]. The combination of lower IL‐17C at 2 years and lower Th22 proportion at 3 months both point towards an immunological shift that could help prevent AD in infancy.

At the 6‐year clinical follow‐up in the ProPACT study, the probiotic group maintained an approximate 40% reduction in the cumulative incidence of AD, although fewer children attended this follow‐up, and the finding was not statistically significant [10, 25]. While our study showed a sustained preventive effect, meta‐analyses suggest that early probiotic supplementation may not lead to long‐term prevention. This could mean that as children grow, environmental and lifestyle factors become more influential, reducing the impact of perinatal probiotics on AD development [5] and Th22 proportions over time.

Despite no clear ongoing link with probiotic supplementation, several Th subsets varied with AD presence and severity. Whilst a smaller study showed no difference in Th22 cells between AD and healthy children between 0 and 5 years [2], the previous analysis of our study suggested an increased proportion of Th22 cells at 3 months in children with AD [8]. In the current study, increased Th22 cells were also observed in children with moderate and severe AD at 2 years, suggesting that IL‐22 cells contribute to the barrier impairment in patients with AD [26, 27]. This is also in line with other studies which have observed associations between IL‐22 produced by Th22 cells and greater transepidermal water loss [2] and AD severity [28, 29]. Since the supplementation did not appear to have earlier or lasting effects on the proportion of Th22 cell in our study, it may not be the primary mechanism behind the prevention of AD. We found no clear differences in other Th subsets or Treg between those with and without AD, though wide confidence intervals prevent ruling out associations. This contrasts with an earlier study linking higher naïve Treg levels to reduced allergic outcomes, including AD [30]. We speculate that higher Treg numbers may prevent AD development, but once AD develops, there may be a compensatory increase in various T cell subsets [30, 31].

The analyses presented in this paper do not provide a conclusive explanation for how maternal probiotic supplementation can lead to the prevention of AD in their offspring. Probiotics may modulate the maternal microbiome, subsequently shaping the infant's microbiome and immune system development [32]. By promoting gut health, probiotic supplementation has been suggested to prevent, manage or alleviate symptoms of AD [33, 34]. In AD, Th2 cells drive inflammation and allergic responses, while Th22 cells, active in both acute and chronic phase, contribute to skin barrier defects and epidermal remodelling [29, 35]. The effect of probiotics in more balanced gut health may reduce systemic inflammation and shifting immune response away from a dominant Th2/Th22 response [6, 36]. This modulation could lead to a more robust skin barrier and a less inflammatory state.

A key strength of this study is the double‐blinded placebo controlled randomised design of the probiotics intervention which followed mother‐infant pairs relatively regularly from birth until 2 years of age. Additionally, the participants showed high compliance with the intervention and avoidance of other probiotics products. The diagnosis of AD was based on validated criteria and families were encouraged to attend the dermatology clinic if the children developed an itchy rash lasting more than 4 weeks to capture all cases of AD.

Whilst this was part of the study protocol for children up to 1 year of age, many families took this opportunity up until 20 months of age. We therefore find it unlikely that cases of AD were undiagnosed, although this is theoretically possible if the symptoms both presented and resolved between 1 and 2 years of age. Another strength of this study is that it also followed the same cohort of children and described T cell development in small children, which in general was rarely conducted previously.

A limitation of this study is the relatively small number of samples available due to incomplete clinical data and or missing samples. Nonetheless, the children included in the this study were similar to the original ProPACT population. The method of measurement for Treg in 3‐month samples was different to that used in the 10‐day and 2‐year samples, resulting in prominent discrepancies, and we therefore opted to compare only 10‐day and 2‐year samples for Treg.

Another limitation of this study is the significant time gap between the analyses conducted in 2015 (3‐month samples), 2018 (10‐day samples) and 2023 (2‐year samples), and within each timepoint the samples were processed in batches. Five separate blood donor samples served as fluorescence‐minus‐one (FMO) controls at the study outset to optimize antibody titres, set compensation, and assess marker expression, with internal controls (known positive/negative populations) used thereafter; no pooled control samples were run across batches (Supporting Information S1). To address batch effects, a post‐hoc statistical assessment of the influence of batches was undertaken using separate linear regression analysis at each timepoint and batch as a categorical variable without any substantial changes in the effect estimates for probiotics supplementation or the development AD (results not shown). While the randomised controlled design reduces the risk of bias between treatment groups, unaddressed technical variation across batches may increase data variability, potentially affecting statistical power and the magnitude or significance of observed effects. Future studies should prioritise methods to assess and correct for batch effects when analyses span extended periods, and with a larger sample size.

The biological interaction is likely to be complex, and this complexity is not assessed with the current analyses. Our analysis was designed to be hypothesis‐driven, focussing on testing pre‐specified outcomes (the effect of probiotic supplementation) using LMM. However, this analysis strategy does not allow a more explorative assessment of the interaction between T cells, probiotic supplementation and AD. Furthermore, in our analyses we have opted to test each T cell subtype separately, rather than attempting to include these in a single model. This decision was made to maintain model interpretability and prevent statistical instability due to high multicollinearity amongst the five biologically interacting T cell subsets (Th1, Th2, Th17, Th22, and Tregs). Future studies could consider more exploratory, unsupervised analyses, such as Principal Component Analysis (PCA) or analyses including specific interactions. PCA could visualise the overall structure of immune variation and identify sources of diversity (like technical or environmental factors) and potentially shed light on the biological complexity not assessed in the current article.

## 5 Conclusion

At the end of maternal probiotic supplementation 3 months after delivery, there was a reduction of Th22 in the probiotic group. However, the supplementation did not appear to have other early or lasting effects on the proportion of Th22cell, and it may not be the primary mechanisms behind the prevention of AD. The presence of AD before the age of 2 years was associated with higher Th22 proportions at 3 months of age, which may persist until 2 years. Children with mild AD had increased proportions of Th22 cells at 3 months of age, and those with moderate to severe AD had increased proportions of Th22 cells at 2 years compared to those without AD. Future studies will need to confirm these apparent associations between Th22 proportions and the presence and severity of AD.

## Author Contributions

**Dinastry Pramadita Zakiudin:** conceptualization, methodology, writing – original draft, writing – review and editing, resources, investigation, project administration, formal analysis, data curation, visualization, software. **Anne Dorthea Bjerkenes Ro:** validation, methodology, supervision, project administration, resources. **Vibeke Videm:** validation, writing – review and editing, supervision, data curation, software. **Gunnhild Vatne Leirvik:** formal analysis, project administration, writing – original draft, resources, software. **Marte Høen Lein:** writing – original draft, formal analysis, project administration, resources, software. **Torbjørn Øien:** supervision, project administration, data curation, funding acquisition, writing – review and editing, validation. **Melanie Rae Simpson:** funding acquisition, visualization, writing – review and editing, supervision, formal analysis, project administration, methodology, validation, data curation, resources, software.

## Conflicts of Interest

The authors declare no conflicts of interest.

## Supporting information


Supporting Information S1

Supporting Information S2

## Data Availability

The data that support the findings of this study are available from the corresponding author upon reasonable request.
