# Supplementary material for: T‐Cell Populations in Infancy After Maternal Probiotic Supplementation to Prevent Atopic Dermatitis
Source: Clin Transl Allergy. 2026 Feb 26;16(3):e70161. doi: 10.1002/clt2.70161 (PMC12945665; doi:10.1002/clt2.70161)
Supplement: Supplementary file 1 — Supporting Information S1 [file CLT2-16-e70161-s001.docx]

**Supplementary file**

Methods, PACT-study 10-days and 2-years samples

# Cell thawing and stimulation

Frozen peripheral blood mononuclear cells (PBMC) were stored in liquid nitrogen and transferred to a -80° C freezer for short time storage (6-12 months). The cells were thawed in a 37° C water bath and gently transferred to centrifuge tubes (15 mL Avantor, VWR, Radnor, PA, USA) with 10 mL RPMI-1640 medium (Cat# R8758-500ML, Sigma Aldrich, St. Louis, MO, USA) and 10% inactivated Fetal Bovine Serum (FBS) (Cat# F7524, Sigma Aldrich, St. Louis, MO, USA). The FCS RPMI solution had room temperature, and the tubes were centrifuged at 400 g for 10 min (21° C). Then, they were decanted, and the cells resuspended in 1 mL FBS RPMI. An ORFLO – Moxi Z Mini Automated Cell Counter (Orflo Technologies, Ketchum, ID, USA) was used to measure cell numbers and viability.

1x10^6^cells/mL were plated into 24 well flat bottom plates (Sarstedt TC-plate 24 well suspension, Sarstedt AG & Co, Nümbrecht, Germany) at 37° C in a humified atmosphere containing 5% CO_2_.

After an overnight rest, cell stimulation Cocktail Plus Transport Inhibitors (500x) (Cat# 00-4975-93, eBioscience, Carlsbad, CA, USA) diluted with FBS RPMI was added to each well to a final volume of 1000 µL or 2000 µL depending on the original volume in each well. The cells were incubated for 5 hours at 37° C, transferred to polystyrene tubes and kept on ice (Falcon round-bottom tube 12x75 mm, BD Bioscience, San Jose, Ca, USA).

# Flow cytometry

The cells were then stained for flow cytometry. Samples in batches of maximum 12, were analysed.

The combination of the 8-colour anti-body panel used:

1. CD3 FITC (Cat# 11-0037-42)
2. IL-4 PE (Cat# 12-7049-42)
3. IL-10 PerCP-eFluor710 (Cat# 46-7108-42)
4. IFNƴ PE-Cy-7 (Cat# 24-7319-82)
5. IL-22 eFluor660 (cat# 50-7229-42)
6. IL-17 APC-eFluor780 (Cat# 47-7179-42)
7. CD4 eFluor450 (# 48-0049-42)
8. Fixable viability dye (FVD) eFluor506 (Cat# 65-0866)

All antibodies were purchased from eBioscience San Diego, CA, USA). Both panel and method were validated in an earlier study (1)

The cells were stained in a three- step protocol, 1) viability staining, 2) surface staining and 3) intracellular (IC) staining:

1. Viability staining

Samples with cell counts 10^5^-10^6^/ tube were washed twice with cold Phosphate-buffered saline (PBS) (Cat# BR0014G, Thermo Fisher Scientific, Wantham, MA, USA). For each washing step throughout the entire protocol, the tubes were centrifuged for 5 minutes at 540 g. After the last wash, the supernatant was discarded and the cells were stained with FVD eFluor506 at 1 µl/ml of cells resuspended in PBS and incubated for 30 minutes at 4 -8 °C, protected from light. The cells were then washed twice with PBS/0, 2 % bovine serum albumin (BSA) (cat# A9418-100G, Sigma Aldrich, St. Louis, MO, USA)/2 mM ethylenediaminetetraacetic acid (EDTA) (cat#108418, Merck, Darmstadt, Germany), pH 7,4. After the last wash, the supernatant was discarded, leaving approximately 50-100 µl residual volume in the tube.

1. Surface staining

Following viability staining, the cells resuspended in the 50-100 µl residual volume, CD3 FITC (5µl) and CD4 eFluor450 (2,5 µl) was added and the tubes were incubated for 30 min at room temperature (RT), protected from light. After incubation, the samples were washed twice with PBS / 0, 2 % BSA / 2 mM EDTA. The supernatant were discarded, leaving approximately 100 µl residual volume in each tube.

1. IC staining:

Prior to IC staining, 100 µl IC Fixation Buffer (Cat# 00-8222, eBioscience San Diego, CA, USA) was added and the tubes were incubated in 30 min at RT, protected from light. Thereafter, the samples were washed with 2 ml 1x Permeabilization Buffer (Cat# 00-8333, eBioscience, San Diego, CA, USA). The supernatant was discarded, and the cells were resuspended in the approximate 100 µl residual volume. Then, IL-4 PE (5µl), IL-10 PerCP-ef710 (5µl), IFNƴ PE-Cy-7 (0,29 µl), IL-22 eFluor660 (5µl), IL-17 APC-eFluor780 (10µl) and CD4 eFluor450 (5µl) were added, and the samples were incubated for 45 min at RT, protected from light. The samples were washed twice with 1x Permeabilization Buffer. After the final washing step, the supernatant was discarded and the cell pellet was resuspended in 300 µl acquisition buffer (BD FACSFlow Cat#342003, BD Bioscience, San Jose, CA, USA) and immediately analysed on a FACSLyric flow cytometer equipped with the FACSSuite software v 1.5 (BD Bioscience, San Jose, CA, USA). For each sample, minimum 100000 events were collected.

Cytometer set-up and compensation were according to EuroFlow guidelines ([www.EuroFlow.org](http://www.EuroFlow.org)). The flow cytometer was calibrated daily using BD Setup and Tracking beads (CS&T Cat# 656505, BD Bioscienses, San Jose, CA, USA).

For data analysis, the Infinicyt software v 2.0 was applied (Cytognos S.L., BD, Salamanca, Spain).

**Controls**

Frozen PBMC separated from five healthy donors, and treated the same way as the study samples, were used as Fluorescence-minus–one (FMO) controls (samples stained with all antibodies in the panel except the one of interest). In addition, internal cell populations were used as controls. Positive events were defined as positive above the level of controls.

**References**

1. Rø ADB, Simpson MR, Rø TB, Storrø O, Johnsen R, Videm V, et al. Reduced Th22 cell proportion and prevention of atopic dermatitis in infants following maternal probiotic supplementation. Clinical & Experimental Allergy. 2017:n/a-n/a.
