# Supplementary material for: T‐Cell Populations in Infancy After Maternal Probiotic Supplementation to Prevent Atopic Dermatitis
Source: Clin Transl Allergy. 2026 Feb 26;16(3):e70161. doi: 10.1002/clt2.70161 (PMC12945665; doi:10.1002/clt2.70161)
Supplement: Supplementary file 2 — Supporting Information S2 [file CLT2-16-e70161-s002.docx]

**Table S1: Comparison of probiotic vs placebo group using non-parametric Mann-Whitney U-test for each Th subset and timepoint**

|  | **Probiotic** | |  | | **Placebo** | |  | **Probiotic vs Placebo^a^: linear mixed model** | | **Probiotic vs Placebo: Mann-Whitney U-test** | |
| --- | --- | --- | --- | --- | --- | --- | --- | --- | --- | --- | --- |
| **Time** | **N** | **Median (IQR)** | | **N** | | **Median (IQR)** | | **Estimated fold-change**  **(95 % CI)** | **p-value** | **p-value** | **Probability** |
| **Th1** |  |  | |  | |  | |  |  |  |  |
| 10 days | 49 | 0.42 (0.33 to 0.61) | | 63 | | 0.45 (0.32 to 0.64) | | 1.00 (0.78 to 1.28) | 1.00 | 1.00 | 0.50 |
| 3 months ^b^ | 37 | 0.80 (0.60to 1.19) | | 39 | | 0.81 (0.53 to 1.14) | | 1.07 (0.79 to 1.44) | 0.67 | 0.59 | 0.46 |
| 2 years | 78 | 2.47 (1.04 to 4.43) | | 78 | | 3.11 (1.57 to 4.15) | | 0.86 (0.69 to 1.07) | 0.17 | 0.14 | 0.57 |
| **Th2** |  |  | |  | |  | |  |  |  |  |
| 10 days | 49 | 0.35 (0.22 to 0.50) | | 63 | | 0.30 (0.19 to 0.49) | | 1.11 (0.81 to 1.52) | 0.52 | 0.41 | 0.45 |
| 3 months ^b^ | 37 | 0.23 (0.14 to 0.33) | | 39 | | 0.19 (0.14 to 0.27) | | 1.11 (0.76 to 1.63) | 0.59 | 0.46 | 0.45 |
| 2 years | 78 | 2.99 (1.46 to 6.33) | | 78 | | 3.05 (1.74 to 7.50) | | 0.93 (0.71 to 1.21) | 0.58 | 0.66 | 0.52 |
| **Th17** |  |  | |  | |  | |  |  |  |  |
| 10 days | 49 | 0.10 (0.04 to 0.16) | | 63 | | 0.09 (0.05 to 0.14) | | 0.92 (0.64 to 1.31) | 0.64 | 0.90 | 0.49 |
| 3 months ^b^ | 37 | 0.21 (0.08 to 0.30) | | 39 | | 0.14 (0.06 to 0.21) | | 1.22 (0.80 to 1.88) | 0.36 | 0.20 | 0.41 |
| 2 years | 78 | 0.07 (0.01 to 0.15) | | 78 | | 0.07 (0.02 to 0.16) | | 0.96 (0.68 to 1.35) | 0.81 | 0.58 | 0.53 |
| **Th22** |  |  | |  | |  | |  |  |  |  |
| 10 days | 49 | 0.01 (0.01 to 0.02) | | 63 | | 0.01 (0.01 to 0.03) | | 0.86 (0.62 to 1.19) | 0.37 | 0.21 | 0.57 |
| 3 months ^b^ | 37 | 0.04 (0.03 to 0.06) | | 39 | | 0.06 (0.04 to 0.09) | | 0.73 (0.49 to 1.08) | 0.11 | **0.01** | 0.67 |
| 2 years | 78 | 0.02 (0.00 to 0.06) | | 78 | | 0.02 (0.00 to 0.05) | | 1.17 (0.84 to 1.64) | 0.35 | 0.95 | 0.50 |
| **Treg** |  |  | |  | |  | |  |  |  |  |
| 10 days | 49 | 0.04 (0.03 to 0.06) | | 63 | | 0.04 (0.03 to 0.06) | | 0.97 (0.77 to 1.23) | 0.88 | 0.84 | 0.51 |
| 2 years | 78 | 0.02 (0.00 to 0.03) | | 78 | | 0.02 (0.00 to 0.05) | | 1.00 (0.85 to 1.17) | 1.00 | 0.46 | 0.53 |

^a^ Differences between probiotic and placebo group were estimated using linear mixed regression models on log-transformed T subset

proportions and the estimated coefficients are therefore fold-change estimates; IQR: inter-quartile range; CI: Confidence Interval. ^b^ The results from the previously published results from samples collected at 3 months were also included in this longitudinal study. Bold number: p-value < 0.05.

**Table S2: Observed proportion of T cell subsets by AD diagnosis and estimated differences between children with and without AD**

|  | **AD** |  |  |  | **No AD** | | |  | **AD versus no AD ^a^** | | | |  |
| --- | --- | --- | --- | --- | --- | --- | --- | --- | --- | --- | --- | --- | --- |
| **Time** | **N** | **Mean (SD)** | **Median (IQR)** | | **N** | **Mean (SD)** | **Median (IQR)** | | **Linear mixed model** | | **Mann-Whitney U-test** | | |
|  |  |  |  |  |  |  |  |  | **Estimated fold-change (95 % CI)** | **p-value** | **p-value** | **Probability** | |
| **Th1** |  |  |  | |  |  |  | |  |  |  |  | |
| 10 days | 30 | 0.57 (0.51) | 0.44 (0.31 - 0.66) | | 75 | 0.51 (0.31) | 0.42 (0.33 - 0.61) | | 1.02 (0.77 - 1.36) | 0.90 | 0.498 | 0.972 | |
| 3 months ^b^ | 21 | 0.98 (0.62) | 0.77 (0.54 - 1.35) | | 55 | 0.96 (0.50) | 0.81 (0.60 - 1.14) | | 0.98 (0.70 - 1.37) | 0.90 | 0.539 | 0.597 | |
| 2 years | 42 | 3.94 (4.06) | 3.17 (1.84 - 4.15) | | 114 | 3.31 (3.52) | 2.45 (1.19 - 4.22) | | 1.26 (0.98 - 1.61) | 0.07 | 0.447 | 0.310 | |
| **Th2** |  |  |  | |  |  |  | |  |  |  |  | |
| 10 days | 30 | 0.33 (0.20) | 0.28 (0.16 - 0.45) | | 75 | 0.40 (0.26) | 0.35 (0.21 - 0.50) | | 0.82 (0.57 - 1.17) | 0.27 | 0.584 | 0.182 | |
| 3 months ^b^ | 21 | 0.24 (0.10) | 0.22 (0.17 - 0.26) | | 55 | 0.22 (0.11) | 0.20 (0.13 - 0.30) | | 1.12 (0.73 - 1.71) | 0.62 | 0.440 | 0.419 | |
| 2 years | 42 | 5.94 (7.65) | 2.61 (1.57 - 5.83) | | 114 | 7.12 (10.57) | 3.07 (1.66 - 7.45) | | 0.90 (0.66 - 1.22) | 0.51 | 0.527 | 0.606 | |
| **Th17** |  |  |  | |  |  |  | |  |  |  |  | |
| 10 days | 30 | 0.12 (0.11) | 0.09 (0.04 - 0.17) | | 75 | 0.11 (0.08) | 0.09 (0.05 - 0.14) | | 0.98 (0.66 - 1.46) | 0.92 | 0.504 | 0.943 | |
| 3 months ^b^ | 21 | 0.19 (0.17) | 0.15 (0.11 - 0.22) | | 55 | 0.19 (0.14) | 0.17 (0.06 - 0.29) | | 0.98 (0.61 - 1.57) | 0.94 | 0.521 | 0.776 | |
| 2 years | 42 | 0.11 (0.12) | 0.08 (0.00 - 0.14) | | 114 | 0.14 (0.43) | 0.07 (0.02 - 0.16) | | 1.12 (0.76 - 1.64) | 0.57 | 0.501 | 0.982 | |
| **Th22** |  |  |  | |  |  |  | |  |  |  |  | |
| 10 days | 30 | 0.02 (0.02) | 0.01 (0.01 - 0.02) | | 75 | 0.02 (0.02) | 0.01 (0.01 - 0.03) | | 0.90 (0.62 - 1.29) | 0.56 | 0.541 | 0.514 | |
| 3 months ^b^ | 21 | 0.10 (0.05) | 0.09 (0.06 - 0.12) | | 55 | 0.05 (0.04) | 0.04 (0.03 - 0.06) | | 2.01 (1.30 - 3.09) | **0.002** | 0.186 | 0.000 | |
| 2 years | 42 | 0.06 (0.15) | 0.03 (0.00 - 0.06) | | 114 | 0.05 (0.19) | 0.02 (0.00 - 0.04) | | 1.40 (0.97 - 2.03) | 0.07 | 0.454 | 0.369 | |
| **Treg** |  |  |  | |  |  |  | |  |  |  |  | |
| 10 days | 30 | 0.05 (0.04) | 0.04 (0.03 - 0.06) | | 75 | 0.05 (0.04) | 0.04 (0.03 - 0.06) | | 0.91 (0.62 - 1.35) | 0.65 | 0.577 | 0.220 | |
| 2 years | 42 | 0.08 (0.34) | 0.02 (0.00 - 0.05) | | 114 | 0.14 (0.83) | 0.02 (0.00 - 0.03) | | 1.00 (0.84 - 1.20) | 1.00 | 0.462 | 0.458 | |

^a^ Differences between AD and no AD group were estimated using linear mixed regression models on log-transformed T subset

proportions and the estimated coefficients are therefore fold-change estimates; AD: Atopic Dermatitis; SD: Standard Deviation; IQR: inter-quartile range; CI: Confidence Interval. ^b^ The results from the previously published results from samples collected at 3 months were also included in this longitudinal study. Bold number: p-value < 0.05.

**Table S3: Observed T cell subsets by AD severity**

|  | **No AD** | |  | **Mild** | |  | **Moderate/Severe** | | **Mild vs No AD^a^** | |  | **Moderate/Severe vs No AD^b^** | |  |
| --- | --- | --- | --- | --- | --- | --- | --- | --- | --- | --- | --- | --- | --- | --- |
| **Time** | **N** | **Mean (SD)** | | **N** | **Mean (SD)** | | **N** | **Mean (SD)** | **Estimated fold-change**  **(95 % CI)** | **p-value** | | **Estimated fold-change**  **(95 % CI)** | **p-value** | |
| **Th1** |  |  | |  |  | |  |  |  |  | |  |  | |
| 10 days | 75 | 0.51 (0.31) | | 15 | 0.67 (0.66) | | 15 | 0.46 (0.27) | 1.15 (0.79 to 1.66) | 0.46 | | 1.11 (0.62 to 1.31) | 0.60 | |
| 3 months ^c^ | 55 | 0.96 (0.50) | | 17 | 0.94 (0.65) | | 4 | 1.16 (0.46) | 1.09 (0.64 to 1.32) | 0.65 | | 1.28 (0.65 to 2.52) | 0.48 | |
| 2 years | 114 | 3.31 (3.52) | | 32 | 3.67 (4.13) | | 10 | 4.79 (3.89) | 1.13 (0.86 to 1.48) | 0.38 | | 1.79 (1.14 to 2.83) | **0.01** | |
| **Th2** |  |  | |  |  | |  |  |  |  | |  |  | |
| 10 days | 75 | 0.40 (0.26) | | 15 | 0.40 (0.20) | | 15 | 0.25 (0.18) | 1.05 (0.66 to 1.69) | 0.83 | | 1.58 (0.39 to 1.02) | 0.06 | |
| 3 months ^c^ | 55 | 0.22 (0.11) | | 17 | 0.24 (0.11) | | 4 | 0.20 (0.04) | 1.14 (0.71 to 1.81) | 0.59 | | 1.03 (0.43 to 2.45) | 0.94 | |
| 2 years | 114 | 7.12 (10.57) | | 32 | 6.00 (7.67) | | 10 | 5.75 (8.02) | 1.11 (0.64 to 1.26) | 0.53 | | 1.09 (0.53 to 1.59) | 0.75 | |
| **Th17** |  |  | |  |  | |  |  |  |  | |  |  | |
| 10 days | 75 | 0.11 (0.08) | | 15 | 0.15 (0.12) | | 15 | 0.09 (0.09) | 1.30 (0.78 to 2.18) | 0.32 | | 1.36 (0.44 to 1.24) | 0.25 | |
| 3 months ^c^ | 55 | 0.19 (0.14) | | 17 | 0.20 (0.18) | | 4 | 0.16 (0.12) | 1.00 (0.60 to 1.67) | 0.99 | | 1.13 (0.34 to 2.29) | 0.81 | |
| 2 years | 114 | 0.14 (0.43) | | 32 | 0.10 (0.11) | | 10 | 0.11 (0.13) | 1.06 (0.70 to 1.60) | 0.79 | | 1.40 (0.65 to 3.04) | 0.39 | |
| **Th22** |  |  | |  |  | |  |  |  |  | |  |  | |
| 10 days | 75 | 0.02 (0.02) | | 15 | 0.02 (0.19) | | 15 | 0.02 (0.02) | 0.84 (0.53 to 1.35) | 0.48 | | 1.05 (0.60 to 1.53) | 0.84 | |
| 3 months ^c^ | 55 | 0.05 (0.04) | | 17 | 0.10 (0.06) | | 4 | 0.09 (0.04) | 2.02 (1.27 to 3.20) | **0.003** | | 1.96 (0.83 to 4.61) | 0.12 | |
| 2 years | 114 | 0.05 (0.19) | | 32 | 0.04 (0.05) | | 10 | 0.16 (0.29) | 1.08 (0.72 to 1.62) | 0.72 | | 3.10 (1.61 to 5.96) | **<0.001** | |
| **Treg** |  |  | |  |  | |  |  |  |  | |  |  | |
| 10 days | 75 | 0.05 (0.39) | | 15 | 0.06 (0.05) | | 15 | 0.04 (0.02) | 1.05 (0.69 to 1.60) | 0.83 | | 1.26 (0.52 to 1.21) | 0.29 | |
| 2 years | 114 | 0.13 (0.82) | | 32 | 0.03 (0.03) | | 10 | 0.25 (0.70) | 1.06 (0.66 to 1.36) | 0.76 | | 1.70 (0.94 to 3.07) | 0.08 | |

^a^ Differences between Mild vs No AD group and ^b^ Moderate/Severe vs No AD group were estimated using linear mixed regression models on log-transformed T subset proportions and the estimated coefficients are therefore fold-change estimates; AD: Atopic Dermatitis; SD: Standard Deviation; CI: Confidence Interval. ^c^ The results from the previously published results from samples collected at 3 months were also included in this longitudinal study. Bold number: p-value < 0.05.

**Table S4: T cell development over time**

| **T-cells and timepoint** | **N** | **Mean (SD)** | **Median**  **(25th - 75th percentile)** | **Range** |
| --- | --- | --- | --- | --- |
| **Th1** |  |  |  |  |
| 10 days | 112 | 0.52 (0.37) | 0.43 (0.32 to 0.61) | 0.08 to 2.93 |
| 3 months ^a^ | 76 | 0.96 (0.53) | 0.80 (0.58 to 1.17) | 0.34 to 2.57 |
| 2 years | 156 | 3.48 (3.67) | 2.70 (1.33 to 4.20) | 0.00 to 27.27 |
| **Th2** |  |  |  |  |
| 10 days | 112 | 0.38 (0.24) | 0.32 (0.20 to 0.50) | 0.08 to 1.31 |
| 3 months ^a^ | 76 | 0.23 (0.11) | 0.22 (0.14 to 0.30) | 0.05 to 0.50 |
| 2 years | 156 | 6.80 (9.86) | 3.02 (1.60 to 7.20) | 0.00 to 61.21 |
| **Th17** |  |  |  |  |
| 10 days | 112 | 0.11 (0.09) | 0.09 (0.04 to 0.16) | 0.00 to 0.41 |
| 3 months ^a^ | 76 | 0.19 (0.15) | 0.15 (0.07 to 0.28) | 0.01 to 0.69 |
| 2 years | 156 | 0.13 (0.37) | 0.07 (0.02 to 0.16) | 0.00 to 4.55 |
| **Th22** |  |  |  |  |
| 10 days | 112 | 0.02 (0.02) | 0.01 (0.01 to 0.02) | 0.00 to 0.08 |
| 3 months ^a^ | 76 | 0.07 (0.05) | 0.05 (0.03 to 0.08) | 0.00 to 0.26 |
| 2 years | 156 | 0.05 (0.18) | 0.02 (0.00 to 0.05) | 0.00 to 2.00 |
| **Treg** |  |  |  |  |
| 10 days | 112 | 0.05 (0.04) | 0.04 (0.03 to 0.06) | 0.01 to 0.28 |
| 2 years | 156 | 0.12 (0.72) | 0.02 (0.00 to 0.04) | 0.00 to 7.63 |

SD: Standard Deviation. ^a^ The results from the previously published results from samples collected at 3 months were also included in this longitudinal study.
